# A clinical decision-making model for repeat surgical treatment of pectus Bar displacement: distance measurement after nuss procedure

**DOI:** 10.1186/s13019-016-0415-1

**Published:** 2016-01-19

**Authors:** Young Jo Sa, Jongho Lee, Jin Yong Jeong, Moonhee Choi, Soo Seog Park, Sung Bo Sim, Keon Hyon Jo

**Affiliations:** Department of Thoracic and Cardiovascular Surgery, Yeouido St. Mary’s Hospital, College of Medicine, The Catholic University of Korea, Seoul, Republic of Korea; Department of Thoracic and Cardiovascular Surgery, Daejeon St. Mary’s Hospital, College of Medicine, The Catholic University of Korea, Seoul, Republic of Korea; Department of Thoracic and Cardiovascular Surgery, Incheon St. Mary’s Hospital, College of Medicine, The Catholic University of Korea, 222 Banpo-daero, Seocho-gu, Seoul, 137-701 Republic of Korea; Department of Anesthesiology, Incheon St. Mary’s Hospital, College of Medicine, The Catholic University of Korea, Seoul, Republic of Korea; Department of Thoracic and Cardiovascular Surgery, St. Paul’s Hospital, College of Medicine, The Catholic University of Korea, Seoul, Republic of Korea; Department of Thoracic and Cardiovascular Surgery, Seoul St. Mary’s Hospital, College of Medicine, The Catholic University of Korea, Seoul, Republic of Korea

**Keywords:** Pectus excavatum, Bar displacement, Minimally invasive surgery, Complication

## Abstract

**Background:**

Bar displacement is one of the most common and serious complications after the Nuss procedure. However, measurements of and factors affecting bar displacement have not been reported. The objectives of this study were to develop a decision model to guide surgeons considering repeat treatment and to estimate optimal cut-off values to determine whether reoperation to correct bar displacement is warranted.

**Methods:**

From July 2011 to August 2013, ninety bars were inserted in 61 patients who underwent Nuss procedures for pectus excavatum. Group A did not need surgical intervention and Group B required reoperation for bar displacement. Bar position was measured as the distance from the posterior superior end of the sternal body to the upper border of the metal bar on lateral chest radiographs. The bar displacement index (BDI) was calculated using D_0_ - D_x_ / D_0_ x 100 (D_0_: bar position the day after surgery; D_x_: minimal or maximal distance of bar position on the following postoperative days). The optimal cut-off values of BDI warranting reoperation were assessed on the basis of ROC curve analysis.

**Results:**

Of the 61 patients, 32 had single bars inserted whereas 29 had parallel bars inserted. There was a significant difference in age (14.0 ± 7.5 vs. 23.3 ± 12.0, *p* = 0.0062), preoperative Haller index (HI) (4.0 ± 1.1 vs. 5.0 ± 1.0, *p* = 0.033), and postoperative HI (2.7 ± 0.4 vs. 3.2 ± 0.5 *p* = 0.006) between the two groups. The optimal cut-off value of BDI was 8.7.

**Conclusions:**

We developed a BDI model for surgeons considering performing reoperation after Nuss procedure. The optimal cut-off value of BDI was 8.7. This model may help surgeons to decide objectively whether corrective surgery should be performed. The main factors affecting the relationship between bar displacement and reoperation were age and preoperative HI.

## Background

The Nuss procedure is used worldwide as a minimally invasive method to repair pectus excavatum (PE) [1]. Bar displacement is one of the most serious complications after the Nuss procedure. Incidence rates of bar displacement vary from 1.8 to 16.6 % [1–5]. Bar displacement may result not only in morphologic changes of the thorax, but can also result in life threatening conditions that require emergency surgery [6]. It occurs most frequently within 30 days following surgery.

Bar displacement can occur through the following three mechanisms: bar flipping, lateral sliding, and hinge point disruption [7]. Several techniques have been applied to prevent bar displacement, including the use of stabilizers, third point of fixation, bar fixation with stainless-steel wire, using a shorter bar, multipoint pericostal fixation, and placement of two bars [7–11].

Displacement of the pectus bar after surgery can be mild to severe. Some authors have measured the degree of bar displacement as the slope angle of the bar position and classified bar displacement as excellent, incomplete, or poor [10, 12]. Bar displacement requiring reoperation is also referred to as “90° or 180° rotation,” “major displacement,” and “significant displacement” [2, 4, 13].

However, measurements of and factors affecting bar displacement have not been examined rigorously. The objectives of this study were to develop a decision model to guide surgeons considering repeat treatment to correct bar displacement and to estimate optimal cut-off values when considering reoperation using receiver operating characteristic (ROC) curves.

## Methods

### Study sample

From July 2011 to August 2013, ninety bars were inserted in 61 patients who underwent Nuss procedures for the treatment of PE. Indications for surgery were: clinical symptoms such as exertional dyspnea, chest discomfort, and growth retardation; Haller index (HI) > 3.25; cardiac deformity compressed by depressed chest wall; and psychological problems. We divided the patients into two groups: Group A (Table 1: category 1, 2) who did not need more surgical intervention; and Group B (Table 1: category 3, 4) who required reoperation for bar displacement. Written informed consent was obtained from each patient prior to surgery. The study was approved by the Institutional Review Board of Incheon St. Mary’s Hospital, College of Medicine, the Catholic University of Korea. All procedures were carried out by one surgeon.

**Table 1:** Grades of pectus bar displacement

Categories	Description
1 (Mild)	There is no displacement of the bar. *Or* Displacement of the bar has occurred but there are no external morphologic changes.
2 (Moderate)	Displacement of the bar and external morphologic changes have occurred but correction of the bar displacement is not necessary.
3 (Severe)	Displacement of the bar and external morphologic changes have occurred and correction of the bar displacement is necessary.
4 (Urgent)	Displacement of the bar has occurred and resulted in compression of the intrathoracic organs, high risk of organ injury, or abnormal EKG. Urgent correction of the bar displacement should be performed.

### Surgical technique

Under general anesthesia with a single endotracheal tube intubation, the patient was placed in a supine position with both arms abducted. The pectus bar size was measured as the distance around the anterior chest wall from one side to the other side of the chest wall on the midaxillary line. The bar was bent according to the morphologic classification of PE and the crane technique was routinely applied [11]. Each tiny skin incision was made on both lateral chest walls. A subcutaneous tunnel and hinge point were made. A thoracoscopic port (MiniPort 2 mm, Tyco Healthcare UK Ltd., Gosport, UK) for a needlescope (2-mm mini fiber telescope, Richard Wolf GmbH, Knittlingen, Germany) was applied cranially from the skin incision along the mid-axillary line with CO_2_ insufflation.

The pectus clamp was introduced through the hinge point. Another 2 mm port for endoscissors was inserted through a tiny skin incision (Richard Wolf GmbH). After completing dissection of the substernal space with endoscissors under direct vision using video assisted thoracoscopy [14], a 20 Fr. chest tube (Argyle thoracic PVC catheter, Covidien llc, Mansfield, MA, USA) was inserted through both hinge points following the pectus clamp. The pectus bar was inserted under the guidance of the chest tube and rotated. After reassessing the external morphology of the anterior chest wall, anterior pericostal fixation of the bar was performed with No. 5 Ethibond (Ethibond Excel, Ethicon Inc., Somerville, NJ, USA) using an endo-needleholder (Olympus Winter & Ibe GmbH, Hamburg, Germany) or Deschamps needle (B. Braun Aesculap AG, Tuttlingen, Germany) under video assisted thoracoscopy [15]. Both lateral fixations were made with fixators. Drainage catheters were inserted into the pleural cavity of one side and the soft tissue layer of the other side if the patient was a child or into the pleural cavities of both sides if the patient was an adult for draining gas and blood.

On the day of surgery, radiologic studies were done several times in the operating room, recovery room, and intensive care unit for the early detection of major complications such as hemothorax, tension pneumothorax, and major bar displacement. Serial chest PA and lateral views were taken daily from the next day onward. The pectus bar was removed 2 years after insertion for patients less than 12 years old, and 2.5 years after insertion for patients between 12 and 18 years old. For patients over 18 years old, the bar was removed 3 years after insertion.

### Measurement of bar displacement

**Fig. 1:**
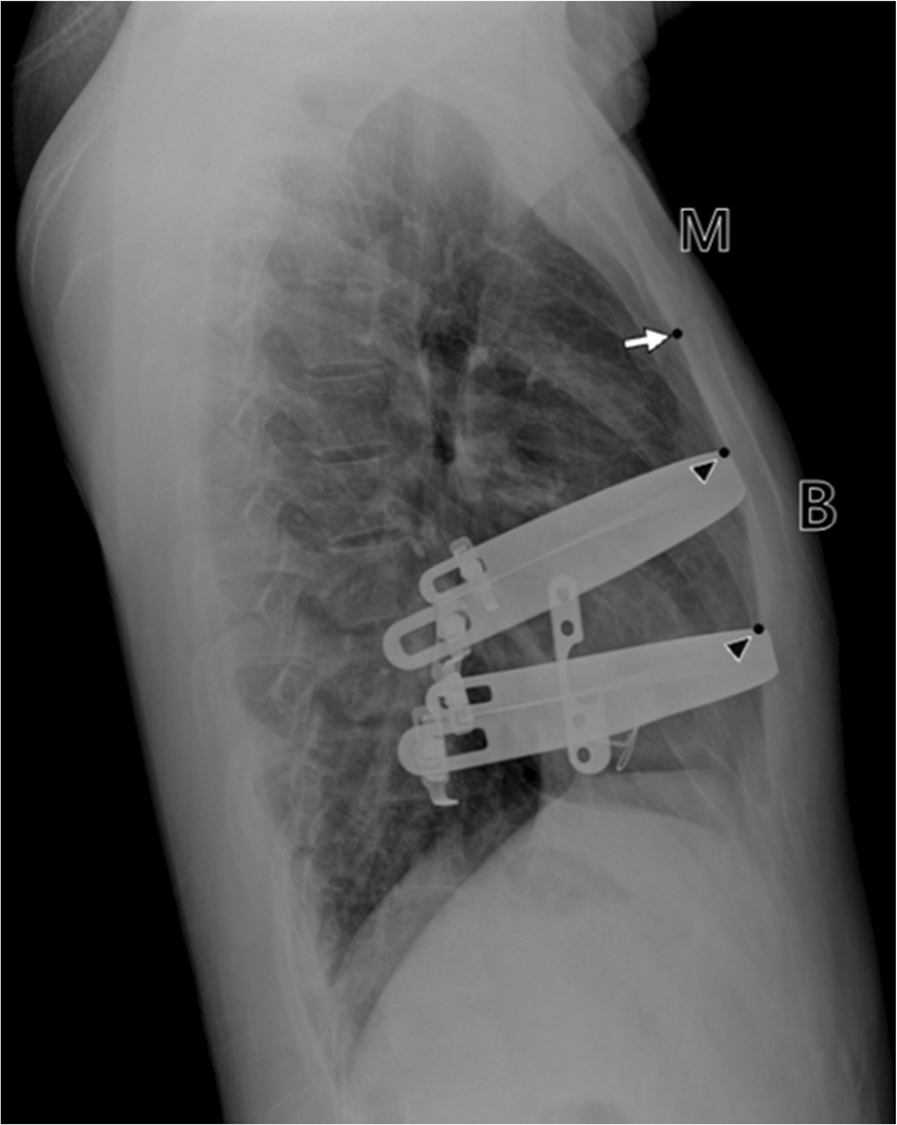
Measurement of bar position. M: manubrium; B: body of the sternum; White arrow: point on the posterior superior end of the sternal body; Black arrow head: point on the superior border of the metal bar

**Fig. 2:**
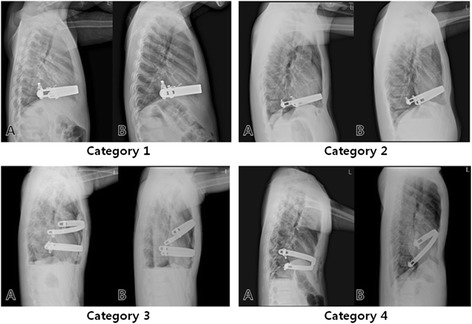
Radiographs describing the four categories of bar displacement. **a** preoperative; **b**: postoperative

Bar position was measured as the distance from the posterior superior end of the sternal body to the upper border of the metal bar on the lateral chest radiograph (Fig. 1). The bar displacement distance was obtained by measuring bar position the day after surgery and the next postoperative days (1 week, 2 weeks, 1 month, and 3 months). The categories of bar displacement were defined as in Table 1 and Fig. 2.

The bar displacement index (BDI) was calculated using the formula of D_0_ - D_x_ / D_0_ x 100, where D_0_ is the bar position the day after surgery, and D_x_ is the minimal or maximal bar position the next postoperative days. In addition, (+) represented upward displacement of the bar, whereas (−) represented downward displacement of the bar.

### Statistical analysis

Continuous and categorical variables were expressed as means ± SD and percentages, respectively. Differences between patients in Group A and Group B were analyzed by Fisher’s exact test for categorical data, and unpaired *t* test for continuous parametric data. Differences between patients in each bar-displacement tertile were assessed by ANOVA or *χ*^2^ tests. Bivariate correlation analysis was utilized to examine the associations between two variables. Statistical significance was defined as a *p* value less than 0.05.

The usefulness of BDI for predicting reoperation was assessed by receiver operating characteristic (ROC) curve analysis. The cut-off values of BDI were defined on the basis of ROC curve analysis by identifying the values of BDI that gave the best combination of sensitivity and specificity. All analyses were performed with SAS 9.3 software.

## Results

### Patient characteristics

Baseline characteristics of patients are summarized in Table 2. There were 7 women and 54 men with a mean age of 15.1 years (range, 3 to 40 years). Of the 61 patients, 32 had single bars inserted, whereas 29 had parallel bars inserted. Of the 90 bars, 42 moved upward postoperatively, 27 moved downward, and 21 moved both upward and downward.

**Table 2:** Patient characteristics and univariate analysis of clinical factors associated with bar displacement

Variable	All (*N* = 61)	Group A (*N* = 54)	Group B (*N* = 7)	*P*
Age	15.1 + 8.6	14.0 + 7.5	23.3 ± 12.0	0.0062
Sex				0.5856
Male	54	47	7	
Female	7	7	0	
BMI	18.4 + 2.8	18.2 ± 2.8	19.5 ± 2.4	0.2582
Height	1.5 + 0.3	1.5 ± 0.3	1.6 ± 0.3	0.4543
Weight	46.8 + 18.6	46.0 ± 18.9	53.3 ± 16.4	0.3361
HI				
Pre- HI	4.1 + 12	4.0 ± 1.1	5.0 ± 1.0	0.033
Post- HI	2.7 + 0.4	2.7 ± 0.4	3.2 ± 0.5	0.0006
Diff- HI	1.4 + 1.1	1.3 ± 1.1	1.7 ± 1.0	0.3175
Type				0.4288
Symmetric	32	27	5	
Asymmetric	29	27	2	
No. of inserted bar				0.2414
1	32	30	2	
2	29	24	5	

There were no significant differences in sex, BMI (height and weight), pectus excavatum type, or the number of bar insertions between Groups A and B (Table 2). There were significant differences in age (14.0 ± 7.5 vs. 23.3 ± 12.0, *p* = 0.0062), preoperative HI (4.0 ± 1.1 vs. 5.0 ± 1.0, *p* = 0.033), and postoperative HI (2.7 ± 0.4 vs. 3.2 ± 0.5 *p* = 0.006) between the two groups. However, HI difference (postoperative HI – preoperative HI) was similar between groups.

### ROC curve analysis of BDI for detecting bar displacement

Clinical characteristics of patients stratified by bar displacement index tertiles are summarized in Table 3. The three tertile groups were comparable in age, sex, pre-HI, post-HI, and type. However, there were significant differences in BMI (weight and height), the number of inserted bars, and grade among the three tertile groups. Patients with higher displacement indexes (tertile 3) were taller and heavier with higher BMI, grade, and number of inserted bars than those in tertile 1 *(p* < 0.05).

**Table 3:** Basic characteristics of bar displacement index tertiles

Variable	|(bar0-bar1)/bar0 | x 100			
	Tertile 1 (0–2.9) N* = 30	Tertile 2 (2.9-7.2) N* = 30	Tertile 3 (>7.2) N* = 30	*P*
Age	14.5 ± 9	15.5 ± 7.6	18.7 ± 7.5	0.1167
Sex				0.6644
Male	22	24	21	
Female	8	6	9	
BMI	18 ± 2.7	18.1 ± 2.9	19.9 ± 2.6	0.0135
Height	1.5 ± 0.3	1.6 ± 0.2	1.7 ± 0.2	0.0141
Weight	43.6 ± 19.4	48.7 ± 16.4	57.9 ± 13.7	0.0049
Pre-HI	4 ± 1.3	4.1 ± 1	4.4 ± 1.2	0.3708
Post-HI	2.6 ± 0.3	2.8 ± 0.4	2.8 ± 0.4	0.0725
Type				0.8747
Symmetric	16(53.3 %)	15(50 %)	17(56.7)	
Asymmetric	14(46.7 %)	15(50 %)	13(43.3)	
Grade				< .0001
1	30(100 %)	25(83.3 %)	13(43.3 %)	
2	0	3(10 %)	9(30 %)	
3	0	2(6.7 %)	5(16.7 %)	
4	0	0	3(10 %)	
No. of inserted bars				< .0.001
1	19(63.3 %)	10(33.3 %)	3(10 %)	
2	11(36.7 %)	20(66.7 %)	27(90 %)	

N* number of bar

**Fig. 3:**
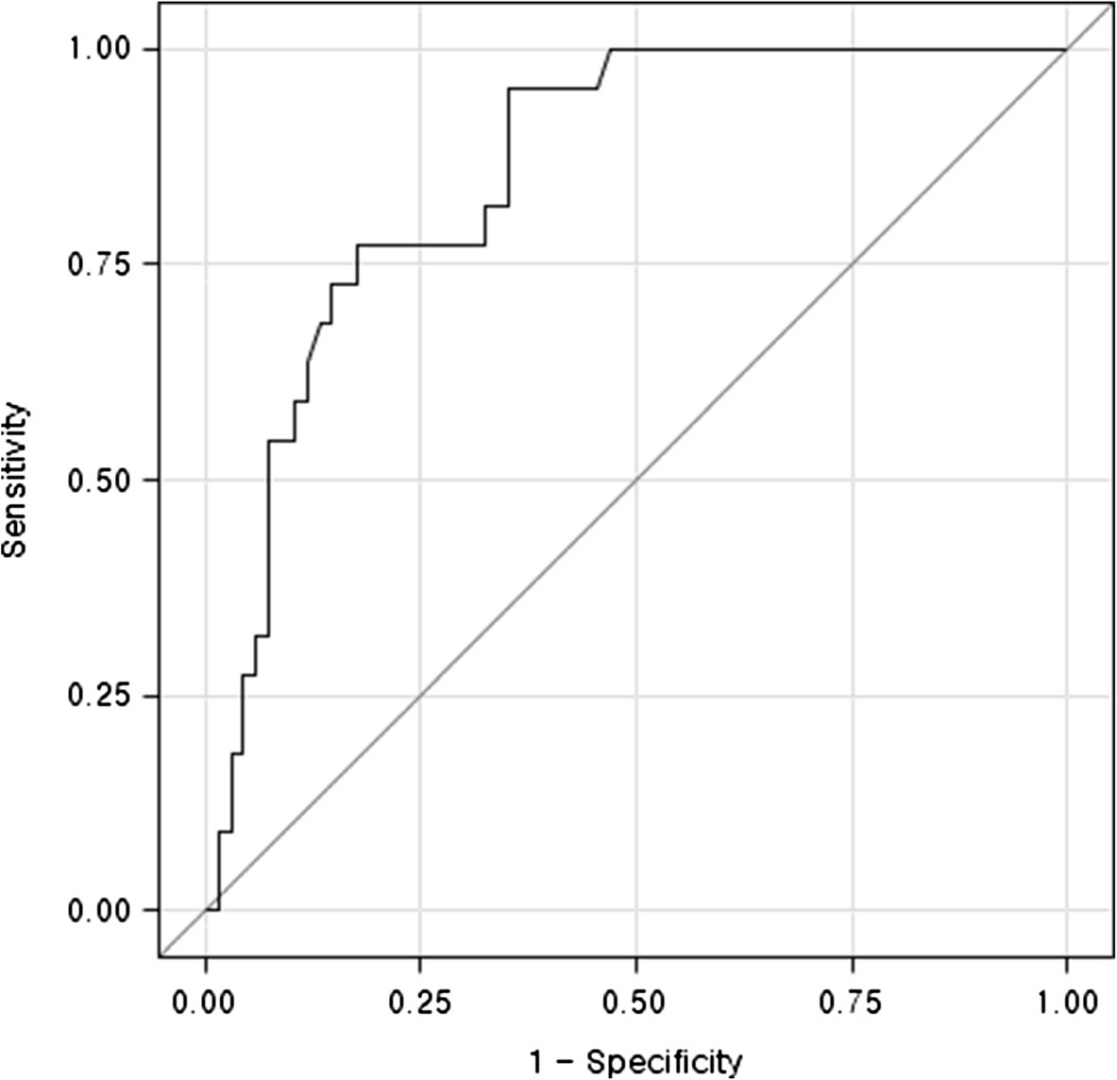
The ROC curve of the bar displacement index for the detection of bar displacement. The area under the curve was 0.858 (95 % CI, 0.769-0.923). The cut-off value of BDI was 8.7

ROC curve analysis was used to assess bar displacement to determine whether surgical repair was necessary. The optimal cut off value of BDI was 8.7. The area under the ROC curve was 0.858 (95 % CI, 0.769-0.923; *p* < 0.0001, Fig. 3).

## Discussion

Park et al. [7] described a variety of mechanisms governing bar displacement, including flipping along the axis of the hinge, sliding laterally to the left or right side, and backward shifting due to stripping of the intercostal musculature. Flipping of the bar is the most common type of displacement [2, 4, 13]. Classifications of bar positions differ between authors. Bar flipping displacement requiring correction is presented in abstract terms such as “90° or 180° rotation,” “major displacement,” and “significant displacement” [2, 4, 13]. Others defined bar positions as excellent (the bar facing the sternum at right angles, less than 15°), incomplete (minimal bar displacement of less than 45°, resulting in minor depression of the sternum), and poor (90° flipped bar with recurred sternal depression) [10, 12].

Displacement of the pectus bar after surgery can be mild to severe. Some authors measure the degree of bar displacement as the slope angle of the bar position. Because these measurements are either obscure or complex, we simply measured the pectus bar position on the lateral projections of chest X-rays (Fig. 1). We defined the BDI as the ratio of the maximal difference between the displacement distance/the distance of the bar position on the day after surgery. ROC curve analysis showed that the cut-off value of BDI indicating a need for corrective surgery for bar displacement was 8.7. Of 61 patients, 46 (75.4 %) and 8 (13.1 %) patients were grades 1 and 2, respectively. These patients did not need surgical correction. Of the remainder (4 patients in grade 3 and 3 patients in grade 4), 5 patients (8.2 %) underwent repeat surgery for correction. Of 4 patients in grade 3, two patients who were satisfied with the external morphological results did not want to undergo reoperation. When the slope angle of the bar was measured in 5 patients with grades 3 and 4 who underwent corrective reoperation, they had bar displacements with angles over 30° (major displacement) [4].

Several devices and techniques have been used to prevent bar displacement [8–11]. We performed not only lateral fixation of the bar, but also anterior pericostal fixation under video-assisted thoracoscopy and the crane technique. This procedure showed a low rate of reoperation to corrent displacement of the pectus bar [16]. Thoracoscopy can display the deepest point of the anterior chest wall during the bar fixation procedure and the position of the bar after completing the procedure, which can reduce the rate of bar displacement as previously mentioned [2]. It also enables safe dissection of the substernal space and identification of the bleeding point and lung entrapment by the bar. However, the application of rigid thoracoscopy may have a disadvantage when showing the opposite side beyond the deepest point in severe pectus excavatum. This problem can be overcome by elevating the depressed anterior chest wall with the use of devices such as the crane technique, suction cups, and sternal lifts [11, 17–19]. Elevating the depressed sternum increases the substernal space, which not only improves the thoracoscopic visual field, but also eliminates the risk of cardiac injury by the dissector, therefore allowing less traumatic placement of the pectus bar.

## Conclusions

We developed a BDI model to help surgeons decide objectively whether reoperation is necessary after the Nuss procedure. The main factors indicating that displaced bars require reoperation are age and preoperative HI.

## Consent

Written informed consent was obtained from all patients or their parents for the publication of this report and any accompanying images.
